# The impact of prolonged landscape fire smoke exposure on women with asthma in Australia

**DOI:** 10.1186/s12884-022-05231-8

**Published:** 2022-12-08

**Authors:** Tesfalidet Beyene, Vanessa E. Murphy, Peter G. Gibson, Vanessa M. McDonald, Joe Van Buskirk, Elizabeth G. Holliday, Anne E. Vertigan, Jay C. Horvat, Graeme R. Zosky, Geoffrey G. Morgan, Edward Jegasothy, Ivan Hanigan, Joerg Mattes, Adam M. Collison, Megan E. Jensen

**Affiliations:** 1 School of Medicine and Public Health, University of Newcastle, Newcastle, NSW Australia; 2 Asthma and Breathing Research Program, Hunter Medical Research Institute, Lot 1 Kookaburra Circuit, New Lambton Heights, Newcastle, NSW 2305 Australia; 3 Department of Respiratory and Sleep Medicine, John Hunter Hospital, Newcastle, NSW Australia; 4 School of Nursing and Midwifery, University of Newcastle, Newcastle, NSW Australia; 5 Sydney School of Public Health, and University Centre for Rural Health, Faculty of Medicine and Health, University of Sydney, Sydney, NSW Australia; 6 Department of Speech Pathology, John Hunter Hospital, Newcastle, NSW Australia; 7 School of Biomedical Sciences and Pharmacy, University of Newcastle, Newcastle, NSW Australia; 8 Menzies Institute for Medical Research, University of Tasmania, Hobart, TAS Australia; 9 Tasmanian School of Medicine, University of Tasmania, Hobart, TAS Australia; 10 John Hunter Hospital and John Hunter Children’s Hospital, Newcastle, NSW Australia

**Keywords:** Asthma, Australia, Landscape fire, Bushfire, Pregnant and/or breastfeeding women, Mental health, Symptoms

## Abstract

**Background:**

Little is known about the physical and mental health impact of exposure to landscape fire smoke in women with asthma. This study examined the health impacts and information-seeking behaviours of women with asthma exposed to the 2019/2020 Australian fires, including women who were pregnant.

**Methods:**

Women with asthma were recruited from the Breathing for Life Trial in Australia. Following the landscape fire exposure period, self-reported data were collected regarding symptoms (respiratory and non-respiratory), asthma exacerbations, wellbeing, quality of life, information seeking, and landscape fire smoke exposure mitigation strategies. Participants’ primary residential location and fixed site monitoring was used to geolocate and estimate exposure to landscape fire-related fine Particulate Matter (PM_2.5_).

**Results:**

The survey was completed by 81 pregnant, 70 breastfeeding and 232 non-pregnant and non-breastfeeding women with asthma. Participants had a median daily average of 17 μg/m^3^ PM_2.5_ and 105 μg/m^3^ peak PM_2.5_ exposure over the fire period (October 2019 to February 2020). Over 80% of participants reported non-respiratory and respiratory symptoms during the fire period and 41% reported persistent symptoms. Over 82% reported asthma symptoms and exacerbations of asthma during the fire period. Half the participants sought advice from a health professional for their symptoms. Most (97%) kept windows/doors shut when inside and 94% stayed indoors to minimise exposure to landscape fire smoke. Over two in five (43%) participants reported that their capacity to participate in usual activities was reduced due to prolonged smoke exposure during the fire period. Participants reported greater anxiety during the fire period than after the fire period (mean (SD) = 53(13) versus 39 (13); *p* < 0.001). Two in five (38%) pregnant participants reported having concerns about the effect of fire events on their pregnancy.

**Conclusion:**

Prolonged landscape fire smoke exposure during the 2019/2020 Australian fire period had a significant impact on the health and wellbeing of women with asthma, including pregnant women with asthma. This was despite most women taking actions to minimise exposure to landscape fire smoke. Effective and consistent public health messaging is needed during landscape fire events to guard the health of women with asthma.

**Supplementary Information:**

The online version contains supplementary material available at 10.1186/s12884-022-05231-8.

## Background

Unprecedented landscape fires (including bushfires, wildfires, and forest fires) have been increasingly reported around the world [1, 2]. The 2019/2020 Australian Black Summer landscape fires (bushfires) resulted in extreme levels of smoke in affected areas for an extended period from October 2019 to February 2020. As a result, most people in Eastern Australia were exposed to poor air quality [2]. Landscape fire smoke causes significantly high concentrations of particulate matter (PM) and gases [2, 3]. Exposure to landscape fire smoke impacts the health of all populations, but some are more vulnerable than others [4]. Women and children, including pregnant and breastfeeding women, and people with existing medical conditions (especially asthma), are at particular risk of being affected by landscape fire smoke [4, 5]. Australia has a high prevalence of people with asthma (11%) [6] and more than 12% of pregnant women have current asthma in Australia [7]. However, there is a lack of information regarding the effect of prolonged landscape fire smoke exposure on women with asthma, including those who are pregnant or breastfeeding.

An association between landscape fire smoke and respiratory morbidity has been reported in multiple studies [3, 8–10]. A study of the impact of prolonged landscape fire smoke exposure in people with severe asthma indicated that 83% of people with severe asthma reported symptoms during the fire period and 65% reported persistent asthma symptoms months after exposure had ceased [11]. Furthermore, studies in Australia found that people with asthma were more likely to report respiratory symptoms and healthcare utilisation, including the use of corticosteroid medications, hospital admission and emergency presentations during the fire period compared to people without asthma [12, 13]. However, previous studies have not specifically focused on women with asthma, including those who are pregnant or breastfeeding.

Environmental factors have a strong influence on in utero development as well as maternal health, with normal physiological changes during pregnancy such as increased cardiac output, minute ventilation and immune changes potentially increasing maternal vulnerability to the effects of extreme air pollution [14–17]. Exposure to landscape fire smoke has been linked with adverse pregnancy outcomes in the general population [18, 19], including decreased birth weight and an increased odds of preterm birth [18, 20].

Landscape fire events may also contribute to mental health problems, such as posttraumatic stress disorder (PTSD) and depression [21–23]. A longitudinal study conducted 3 to 4 years after the Victorian Black Saturday landscape fire in Australia (2009) found that people in highly affected areas had a higher rate of PTSD and depression than those from less affected areas [24]. A recent study of the impact of the 2019/2020 Australian fire on physical and mental health in the Australian Capital Territory found that more than half of the participants reported symptoms of anxiety and/or feeling depressed because of smoke exposure [25]. Notably, women were more likely to report symptoms of anxiety (54% versus 32%) and depression (25% versus 15%) than men, highlighting the vulnerability of this population group. Therefore, investigation into the impact of the 2019/20 Australian landscape fires on the physical and mental health of women with asthma, including those who were pregnant is warranted.

The aim of this study was to examine the physical and mental health impact of prolonged landscape fire smoke exposure on women with asthma, including a group who were pregnant during the fire period, and to characterize women’s health-seeking behaviours and actions to mitigate the impacts of landscape fire smoke exposure.

## Methods

### Study design and setting

We conducted a cross-sectional, retrospective study to assess the impacts of landscape fire smoke exposure in women with asthma during the 2019/2020 Australian Black Summer landscape fire period (1 October 2019 to 29 February 2020).

### Participants

Participants were sourced from the Breathing for Life Trial (BLT) [26, 27] and Breathing for Life Trial-Nutrition in Pregnancy (BLT-NUT) studies of asthma in pregnancy, conducted from 2013 to 2019. Pregnant women aged > 18 years, with physician-diagnosed asthma, and symptoms of asthma or use of asthma pharmacotherapy (β2-agonist, and/or inhaled corticosteroid (ICS) in the past 12 months), and between 12 and 23 weeks gestation was enrolled into a randomised controlled trial of asthma management during pregnancy, with postpartum follow-up and ongoing follow-up of their children. Six sites across Eastern Australia participated in BLT: John Hunter Hospital, Newcastle (NSW); Royal Hospital for Women Randwick, Royal North Shore Hospital, and Nepean Hospital, Sydney (NSW); Royal Brisbane and Women’s Hospital, Brisbane (QLD); and The Canberra Hospital, Canberra (ACT). In 2020, BLT and BLT-NUT participants were contacted and invited to participate in this survey study.

Participants were classified into one of three groups. (a) Women who reported being pregnant during the fire period and either gave birth during the fire period or prior to 20 November 2020 (based on baby’s date of birth, or estimated date of birth, provided in the survey,) were identified as being pregnant. Some of these women were also feeding an infant/toddler during the fire period, therefore, this group was defined as ‘pregnant +/- feeding an infant/toddler’. (b) Women who reported breastfeeding their infant/toddler (< 2 years of age) during the fire period were classed as ‘breastfeeding only’. (c) Women who did not report being pregnant or breastfeeding/feeding an infant/toddler during the fire period were defined as ‘non-pregnant and non-breastfeeding’.

### Data collection tool and technique

Following review of relevant literature and investigator/stakeholder input, a study-specific close ended item survey was designed to capture health outcome(s) experienced, and health seeking behaviour(s), during the landscape fire period [11, 13, 28–31]. A template of the tool was prepared using REDCap (Research Electronic Data Capture) to capture survey responses [32]. The data were collected via a self-report survey of participants following the 2019/2020 Australian fire period, by telephone, online or on paper (Supplementary file 2). The survey commenced on the 19th of May 2020 and closed on the 2nd of December 2020.

Participant demographics, general health, smoking history, and place of residence during the fire period were collected. Participants were asked about general and respiratory health outcomes experienced during and following the fire period, actions they undertook to minimise or avoid exposure to landscape fire smoke and whether they believed these actions were useful in reducing exposure to landscape fire smoke. Participants were asked about the impacts of prolonged landscape fire smoke exposure on their quality of life, with questions from the Asthma Australia bushfire survey [31].

The Six-Item Short-Form State-Trait Anxiety Inventory (STAI-6) was used to retrospectively assess state anxiety during the fire period and following the fire period (i.e., at the time of the survey). The STAI-6 is highly correlated with the full 20-item STAI version [28, 29] and uses a four-point Likert scale scoring system (1 = Not at all, 2 = Somewhat, 3 = Moderately so, 4 = Very much so), with higher total scores indicating greater anxiety. The participants were asked to rate each of the anxiety symptoms during the fire period and at the time of the survey. The total summed scores for anxiety during and after the fire period were pro-rated (multiplied by 20/6) to obtain scores comparable to the full 20-item STAI, which ranges from 20 to 80 [28]. The total STAI-20 score was graded for low [20 - 39], moderate [40 - 59] and high anxiety [> 59] [33]. A cut off point 39-40 on the STAI may indicate clinically significant symptoms for anxiety [34, 35], with previous Australian studies classifying women scoring > 40 as ‘highly anxious’ [36, 37].

Participants’ subjective psychological distress in relation to the fire period was assessed using the Impact of Events Scale-Revised (IES-R) [30]. The IES-R comprises 22 items rated on a 5-point Likert scale (0 = Not at all, 1 = A little bit, 2 = Moderately, 3 = Quite a bit and 4 = Extremely) with respect to how distressing each item has been during the past week [30]. The IES-R items are categorized into three subscales: intrusion (eight items), avoidance (eight items) and hyperarousal (six items). The total IES-R score was graded for normal [0 - 23], mild [24 - 32], moderate [33 - 36] and severe psychological impact [37 - 88]. A cut-off score of 24 was used to identify PTSD of clinical concern [38, 39].

### Landscape fire smoke exposure data

The exposure period was defined as 1 October 2019 to 29 February 2020 (the 2019/2020 black summer fire period in Australia). We obtained daily 24-hour mean fine particulate matter (particulate matter of 2.5 μm or less in diameter, PM_2.5_) data from fixed-site government air quality monitoring stations within the greater Sydney and Melbourne metropolitan regions (NSW Department of Planning, Industry and Environment and Environmental Protection Agency Victoria) and identified landscape fire smoke days from a database based on government data and satellite imagery [40–43]. The measured daily data were interpolated within study regions using an inverse distance weighting procedure to estimate daily PM_2.5_ (μg/m^3^) exposure concentration for participants’ residential locations [44].

Landscape fire days were defined as days when: the regional 24-hour average of PM_2.5_ concentration exceeded the 95th percentile (based on the period 28/01/2014 to 31/12/2018 for the Melbourne region and 01/01/2000 to 31/12/2018 for the Sydney Greater Metropolitan Region (GMR)), and there was visual confirmation of fire for that day, or up to 3 days before or after, via satellite imagery. Elevated PM_2.5_ levels on these days could therefore be attributed to landscape fire smoke [44]. To control for spatial variability in fire smoke in the region, an additional requirement was that the interpolated PM_2.5_ reading for each participant’s residential address also exceeded the 95th percentile for the region.

For each participant, daily PM_2.5_ concentration levels over the 152-days of the fire period were averaged to obtain their mean PM_2.5_ (μg/m^3^) exposure. The participant’s peak PM_2.5_ (μg/m^3^) was determined as the maximum 24-hour concentration value to which a participant was exposed during the 152-day fire period. Total fire days and maximum consecutive fire days were also calculated over the 152-days period.

### Statistical analysis

Data for continuous variables were summarized using mean with standard deviation (SD) or median with interquartile range (Q1, Q3). Data for categorical variables were summarised using frequency with percent. McNemar’s test were used to compare women’s symptoms, and paired t-test was used to compare anxiety scores, during and after the fire period. Statistical analysis was performed using STATA version 16 (Texas, USA). A *p*-value of < 0.05 was considered statistically significant. Figures were prepared using the R package “ggplot2” [45].

## Results

**Fig. 1:**
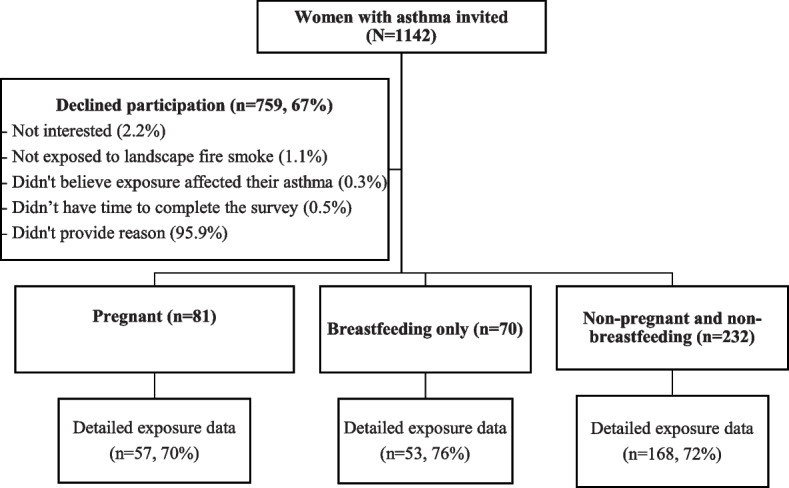
Participant flow diagram

Of the 1142 women with asthma who were invited to participate, 383 (33%) completed the survey. Of these, 81 (21%) women were classed as ‘pregnant’, 70 (18%) as ‘breastfeeding’, and 232 (61%) as ‘non-pregnant and non-breastfeeding’ during the fire period (Fig. 1). Of the 81 pregnant women, 32 (39%) were feeding their infant/toddler during the fire period. Of the 278 women with exposure data, 276 (99%) resided in Sydney GMR and 2 (1%) resided in Melbourne. Surveys were completed a median 5 months (range 3–6 months) after the fire period.

### Landscape fire smoke exposure

Mean daily PM_2.5_ concentrations and fire days in the Sydney GMR and Melbourne region during the 2019/2020 fire period are shown in Fig. S1. During the period from 1 October 2019 to 29 February 2020, there were 55 and 6 identified fire days for Sydney GMR and Melbourne region, respectively. Participants experienced a median daily average PM_2.5_ exposure over the fire period of 16.6 μg/m^3^ [16.4,16.7] and median peak PM_2.5_ of 104.6 μg/m^3^ [100.3108.3]. Participants were exposed to a median of 42 fire days and 11 maximum consecutive fire days (Table 1).

**Table 1:** Landscape fire exposure data of participants

Variables	Median [Q1, Q3]
Average PM_2.5_ (ug/m^3^)	16.6 (16.4, 16.7)
Peak PM_2.5_ (ug/m^3^)	104.6 (100.3108.3)
Total fire days	42 (41,43)
Maximum consecutive fire days	11 (11,11)

### Demographic and general health characteristics

Participants had a mean (SD) age of 34 (5.5) years. Over 63% had never smoked and 45% rated their general health as ‘Good’. Data are presented by groups in Table 2.

**Table 2:** Characteristics of participants at the time of survey

Variables	Pregnant women (***n*** = 81)	Breastfeeding women (***n*** = 70)	Non-pregnant and non-breastfeeding women (***n*** = 232)
Age (years) Mean (SD)	32.2 (5.6)	33.6 (5.3)	34.8 (5.4)
Smoking status n (%)			
Never smoker	55 (67.9)	49 (70.0)	134 (59.3)
Ever smoker	21 (25.9)	17 (24.3)	69 (30.5)
Current smoker	5 (6.2)	4 (5.7)	23 (10.2)
General Health n (%)			
Excellent	5 (6.2)	7 (10.0)	13 (5.6)
Very good	32 (39.5)	29 (41.4)	60 (25.9)
Good	33 (40.7)	28 (40.0)	113 (48.7)
Fair	9 (11.1)	6 (8.6)	36 (15.5)
Poor	2 (2.5)	0 (0.0)	10 (4.3)
Diagnosed with any new condition in the past 6 months n (%)	18 (22.5)	14 (20.0)	37 (16.0)
Had to leave residence or be evacuated during landscape fire period n (%)	2 (2.5)	2 (2.9)	3 (1.3)

*SD* Standard deviation

### Self-reported symptoms during and following landscape fire period

**Fig. 2:**
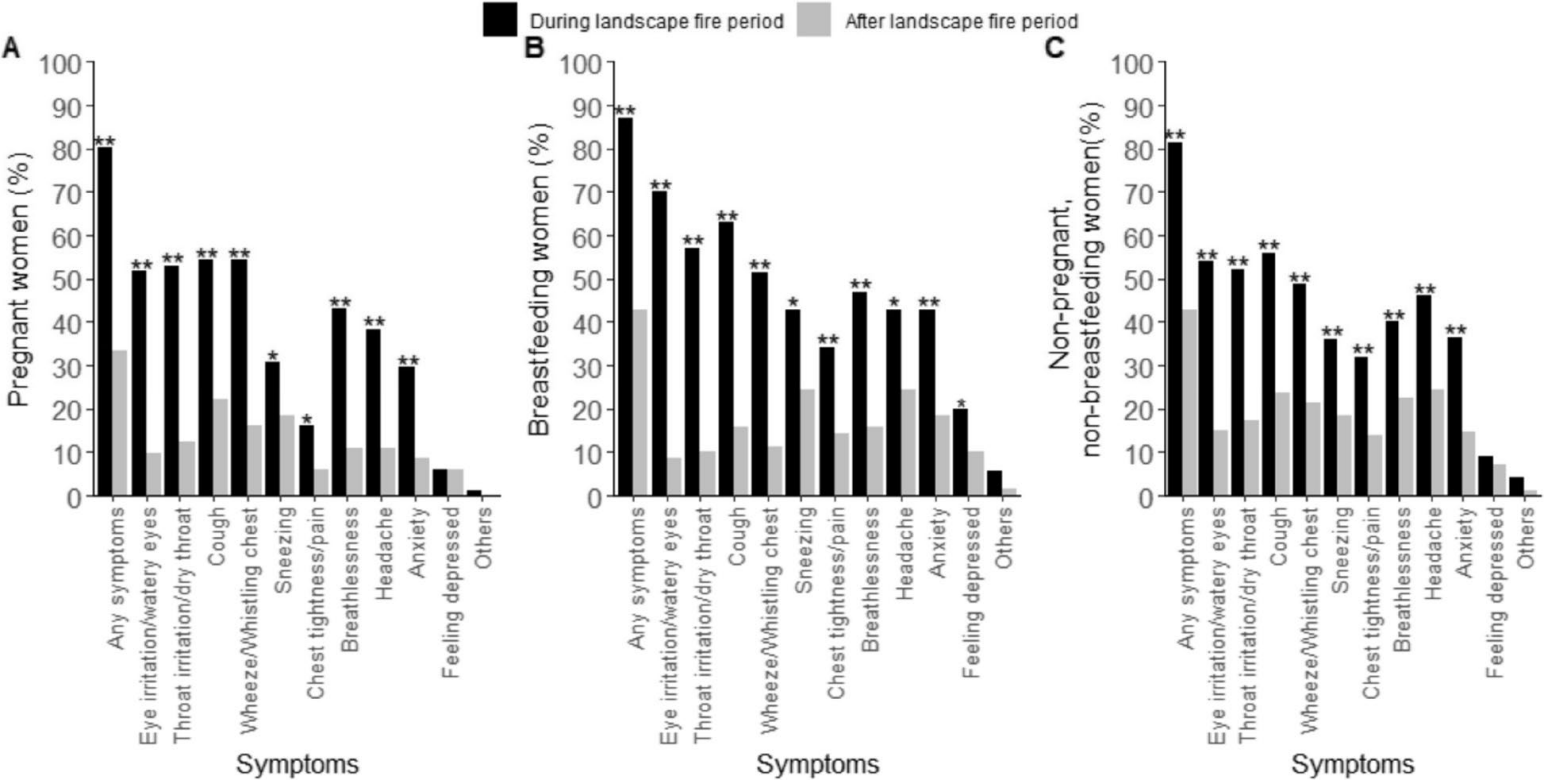
Symptoms reported by participants during and following the 2019/20 Australian Black Summer landscape fire period. (**A**) pregnant, (**B**) breastfeeding, or (**C**) neither pregnant nor breastfeeding. *indicates statistically significant difference in symptom during versus following the fire period. ** *p* < 0.001, * *p* < 0.05

Three-hundred-and-fifteen (82%) participants reported symptoms during the fire period. The most prevalent symptoms were cough (57%), eye irritation/watery eyes (56%), throat irritation/dry throat (53%), and wheeze or whistling chest (50%). Two in five (41%) participants reported symptoms after the fire period (i.e. at the time of survey completion) with cough (22%), headache (22%) and sneezing (20%) the most frequently reported. Statistically significant differences were observed between symptoms during and following the fire period (*p* < 0.001) (combined groups, Fig. S2). Data are presented by group in Fig. 2.

### Asthma symptoms during the landscape fire period

Most (85%) participants reported asthma symptoms during the fire period. Three-hundred-and-twenty-eight (86%) participants reported having an asthma exacerbation during the fire period, with 20% reporting having started/increased OCS use for an asthma exacerbation(s) during the fire period. No participants reported a hospital admission(s) (combined groups, Table S1). Data are shown by group in Table 3.

**Table 3:** Self-reported asthma symptoms during the fire period experienced by participants

Variable	Pregnant (***n*** = 81)	Breastfeeding (***n*** = 70)	Non-pregnant, non-breastfeeding (***n*** = 232)
Experienced asthma symptoms during the landscape fire period, n (%)	71 (87.6)	63 (90.0)	192 (82.8)
Exposure to smoke was the main reason for asthma symptoms, n (%)			
Yes	64 (90.1)	54 (85.7)	159 (82.8)
No	1 (1.4)	4 (6.3)	2 (1.0)
Don’t know/Unsure	6 (8.4)	5 (7.9)	28 (14.5)
Not exposed to landscape fire	0 (0.0)	0 (0.0)	3 (1.6)
Asthma exacerbation	72 (88.9)	65 (92.9)	191 (82.3)
Types of asthma exacerbation, n (%)			
Emergency department	5 (6.2)	1 (1.4)	9 (3.9)
Unscheduled doctor visit	23 (28.4)	20 (28.6)	62 (26.7)
Start/increase of OCS at least 3 days	12 (14.8)	12 (17.1)	53 (22.8)
IV corticosteroids	1 (1.2)	0 (0.0)	4 (1.7)
Increased reliever use	67 (82.7)	61 (87.1)	185 (79.7)
Increased preventer dose/frequency	45 (55.6)	45 (64.3)	119 (51.3)
Number of times, median (Q1, Q3)			
Emergency department	1 (1,1)	2 (2,2)	1 (1,1)
Unscheduled doctor visit	2 (1,3)	2 (1,2.5)	2 (1,3)
Start/increase of OCS ≥3 days	1 (1,4)	1.5 (1,5)	2 (1,3)
IV corticosteroids	NA	NA	1 (0.5,2.5)
Thinks smoke from the landscape fires was the main reason for the exacerbation, n (%)	63 (87.5)	56 (86.1)	166 (86.9)
Not exposed	1 (1.4)	2 (3.1)	5 (2.6)

### Source of information/advice on symptoms, asthma management and minimising exposure to landscape fire smoke

One-hundred-and-fifty-four (49%) participants sought advice from a health professional for their symptoms, of which 37% sought advice from their general practitioner. One-hundred-and-forty-eight (39%) participants reported receiving information/advice on asthma management during the fire period, of which 72% received information from a general practitioner. One hundred-and-seventy-four (45%) participants received advice on avoiding exposure to landscape fire smoke; news/current affairs stories (51%), general practitioner (40%) and social media (40%) were the most frequently reported information sources (combined groups, Table S2). Data are presented by group in Table 4.

**Table 4:** Source of information/advice on symptoms, asthma management and minimising exposure to landscape fire smoke reported by participants during the 2019/20 Australian landscape fires

Action taken	Pregnant women	Breastfeeding women	Non-pregnant and non-breastfeeding
	n (%)	n (%)	n (%)
Sought health advice from a health professional for symptoms	35/65 (53.8)	32/61 (52.5)	87/189 (46.0)
General practitioner	26 (40.0)	23 (37.7)	69 (36.5)
Pharmacist	5 (7.7)	16 (26.2)	35 (18.5)
Other medical professional	15 (23.1)	2 (3.3)	2 (1.1)
Emergency department	1 (1.5)	1 (1.6)	7 (3.7)
24-hour health advice hotline	0 (0.0)	2 (3.3)	6 (3.2)
Hospital inpatient	0 (0.0)	2 (3.3)	0 (0.0)
Took time off work because of the symptoms	17 (26.1)	20 (32.8)	48 (25.4)
Landscape fire was the main reason for any symptoms			
Yes	58 (89.2)	47 (77.0)	156 (82.5)
No	3 (4.6)	4 (6.6)	8 (4.2)
Do not know/unsure	4 (6.1)	10 (16.4)	21 (11.1)
Not exposed to wildfire	0 (0.0)	0 (0.0)	4 (2.1)
Advice on asthma management	34 (42.0)	25 (35.7)	89 (38.4)
General practitioner	25 (73.5)	19 (76.0)	63 (70.8)
Pharmacist	2 (5.9)	7 (28.0)	25 (28.1)
News/current affairs stories	0 (0)	4 (16.0)	26 (29.2)
Respiratory/asthma specialist	11 (32.3)	3 (12.0)	5 (5.6)
Social media	0 (0)	3 (12.0)	14 (15.7)
Nurse	6 (17.6)	1 (4.0)	5 (5.6)
Family/friends	1 (2.9)	2 (8.0)	6 (6.7)
Health department	0 (0)	1 (4.0)	7 (7.9)
Asthma Australia	0 (0)	3 (12.0)	4 (4.5)
Midwife/Obstetrician	5 (14.7)	1 (4.0)	NA
Others^a^	4 (11.8)	4 (16.0)	4 (4.5)
Received advice about how to avoid or minimise exposure to landscape fire smoke	36/81 (44.4)	32/70 (45.7)	106/232 (45.7)
News/current affairs stories	12 (33.3)	18 (56.2)	58 (54.7)
General practitioner	17 (47.2)	14 (43.7)	38 (35.8)
Social media	9 (25.0)	12 (37.5)	48 (45.3)
Family/friends	4 (11.1)	9 (28.1)	17 (16.0)
Health department	5 (13.9)	5 (15.6)	19 (17.9)
Pharmacist	2 (5.6)	3 (9.4)	15 (14.1)
Respiratory/asthma specialist	8 (22.2)	1 (3.1)	3 (2.8)
Asthma Australia	3 (8.3)	2 (6.2)	6 (5.7)
Nurse	4 (11.1)	0 (0)	3 (2.8)
Midwife/Obstetrician	5 (13.9)	0 (0)	NA
Others^a^	5 (13.9)	5 (15.6)	11 (10.4)

^a^clinical research assistance (nurse), support group, 24-hour health advice hotline, other medical professionals, *NA* Not applicable

### Landscape fire smoke exposure risk mitigation

**Fig. 3:**
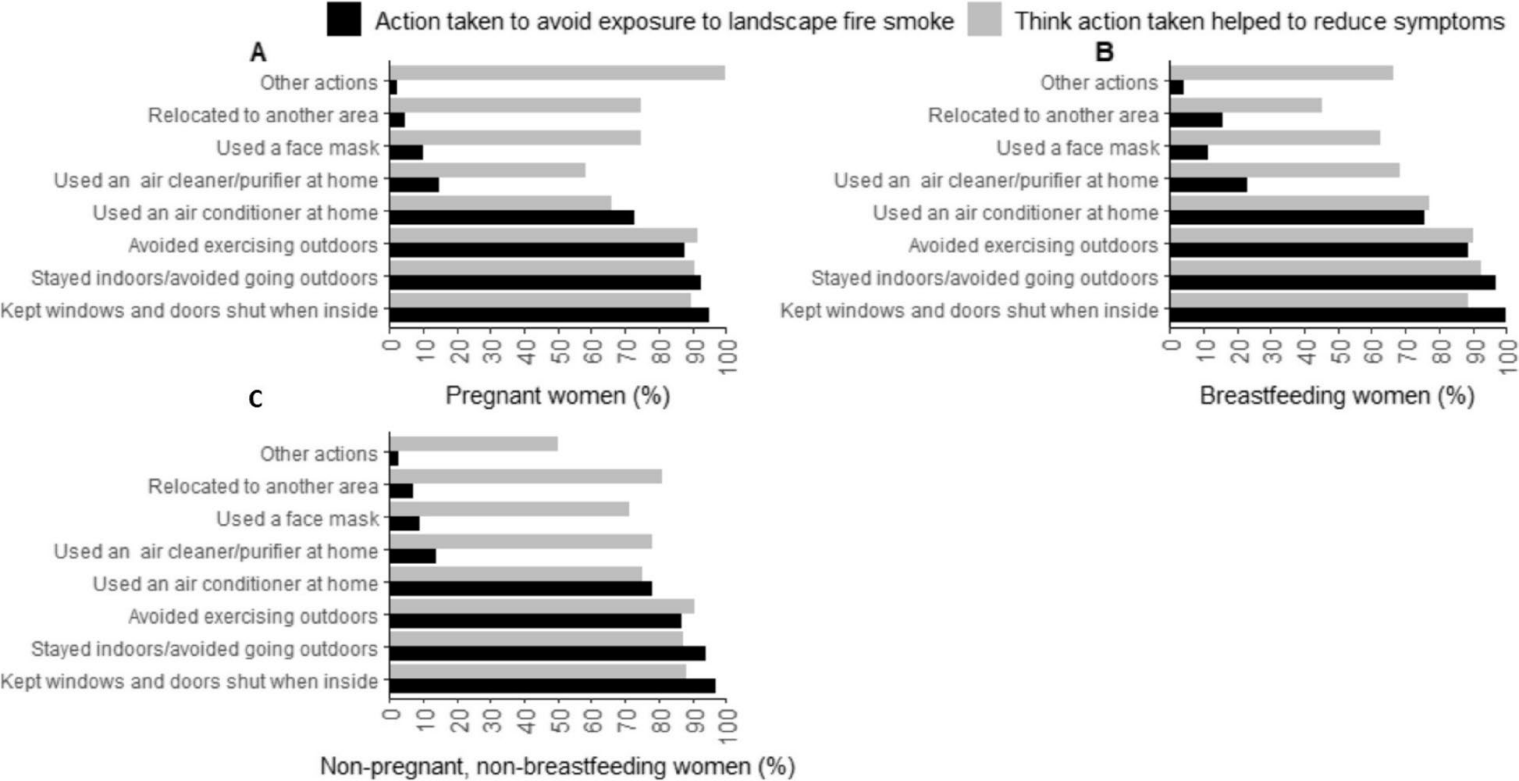
Actions taken by participants with asthma to minimise exposure to landscape fire smoke during the 2019/20 Australian landscape fires. (**A**) pregnant, (**B**) breastfeeding, or (**C**) neither pregnant nor breastfeeding

Most (97%) participants reported keeping windows and doors shut when inside and 94% stayed indoors/avoided going outdoors to minimise exposure to landscape fire smoke. Most (91%) thought avoiding exercising outdoors helped to reduce symptoms (combined groups, Fig. S3). Data are shown by group in Fig. 3.

### Impact of landscape fire smoke exposure on quality of life

**Fig. 4:**
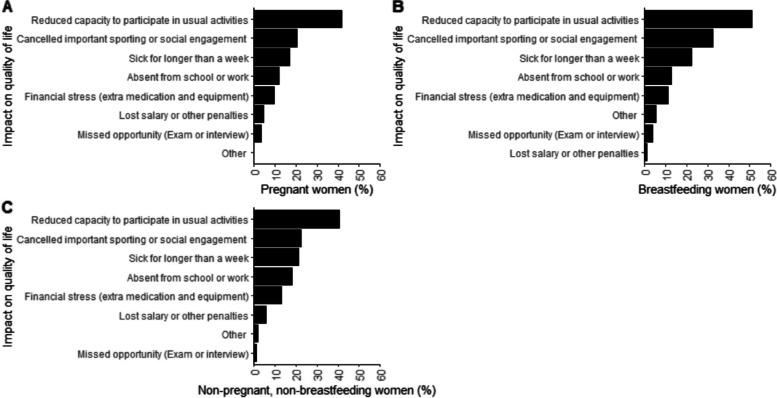
Impact of prolonged smoke exposure from the 2019/20 Australian landscape fires on quality of life in women with asthma, who were (**A**) pregnant, (**B**) breastfeeding, or (**C**) neither pregnant nor breastfeeding

Forty-three percent of participants reported that prolonged landscape fire smoke exposure reduced their capacity to participate in usual activities during the fire period and 24% reported cancelling important sporting or social engagements (combined groups, Fig. S4). Data are presented by group in Fig. 4.

### Anxiety, during and following the 2019/2020 Australian landscape fire period and the psychological impact during fire period

**Fig. 5:**
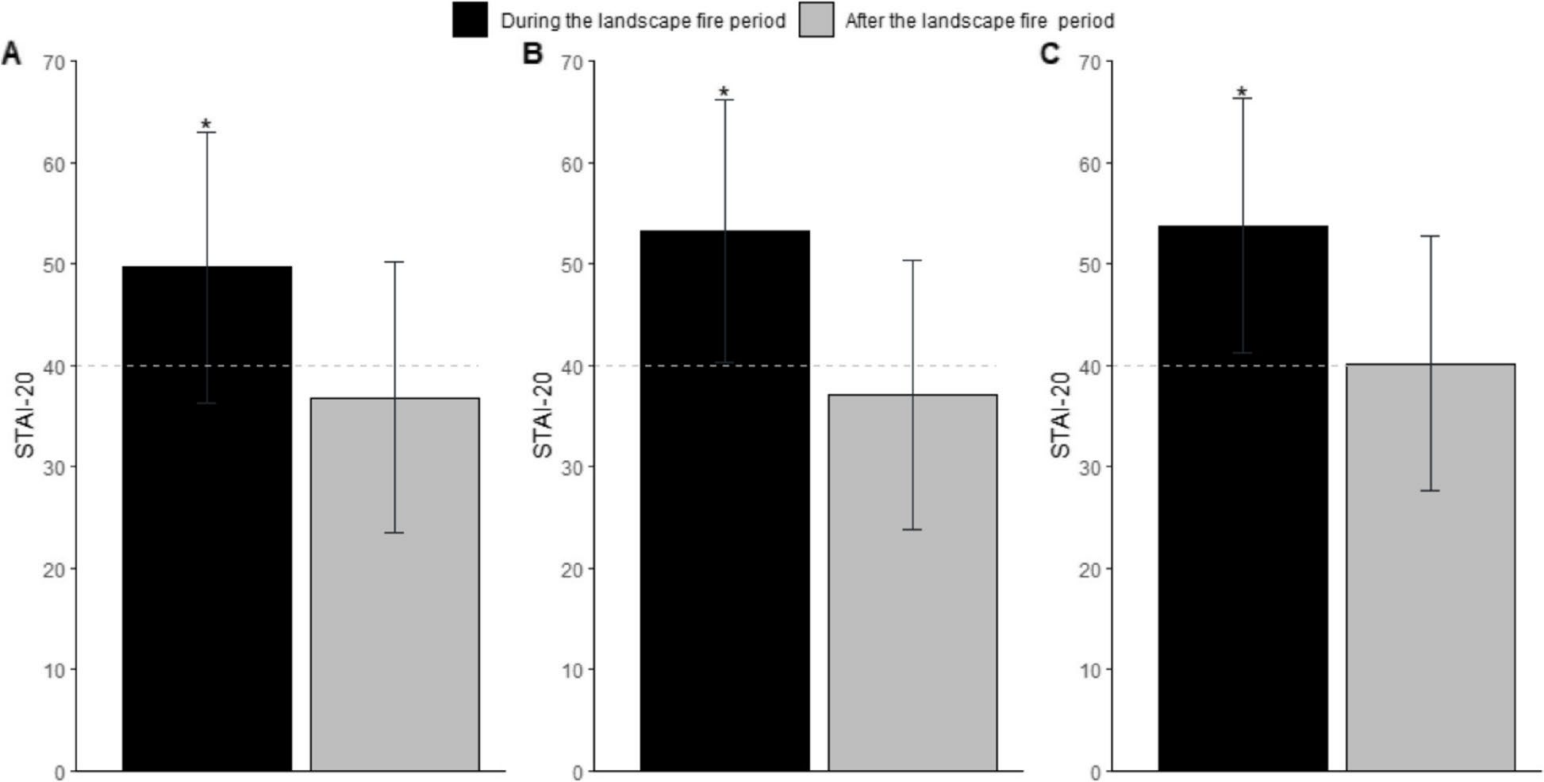
Mean anxiety score amongst participants during, and after, the 2019/20 Australian landscape fires. (**A**) pregnant, (**B**) breastfeeding, or (**C**) neither pregnant nor breastfeeding The horizontal dashed line indicates a cut-off point for clinically significant symptoms of anxiety using the STAI-20. Error bars indicate the standard deviation. *indicates statistically significant difference in anxiety during versus following the fire period [*p* < 0.001]. STAI, State-Trait Anxiety Inventory

Self-reported anxiety levels were higher during the fire period, compared to after the fire period (mean (SD) = 52.9 (12.9) versus 38.9 (12.9), *p* < 0.001) (combined groups Fig. S5). This was also true when examining STAI score by group (Fig. 5, Table 5), with significantly more participants classed as having high anxiety before, vs after, the bushfire period (Table 5).

**Table 5:** Anxiety experienced by participants during and following the 2019/20 Australian landscape fires and the psychological impact

Variables	Pregnant women (***n*** = 81)	Breastfeeding women (***n*** = 70)	Non-pregnant and non-breastfeeding women (***n*** = 232)
	Value	Value	Value
Anxiety score (STAI-6) during landscape fire period, n (%)			
Low anxiety (20–39)	21 (25.9)	8 (11.4)	25 (10.8)
Moderate anxiety (40–59)	40 (49.4)	39 (55.7)	129 (55.6)
High Anxiety (60–80)	20 (24.7)	23 (32.9)	78 (33.6)
Anxiety score (STAI-6) following the landscape fire period, n (%)			
Low anxiety (20–39)	48 (59.3)	42 (60.0)	105 (45.3)
Moderate anxiety (40–59)	26 (32.1)	23 (32.9)	107 (46.1)
High Anxiety (60–80)	7 (8.6)	5 (7.1)	20 (8.6)
IES-R median (Q1, Q3) Total sum score (0–88),	1 (0,6)	2 (0,7)	2 (0,8)
Sum Intrusion score (0–32)	1 (0,2)	1 (0,3)	1 (0,3)
Sum Avoidance score (0–32)	0 (0,3)	1 (0,4)	1 (0,4)
Sum Hyperarousal score (0–24)	0 (0,0)	0 (0,1)	0 (0,1)
Total IES-R score, n (%)			
Normal (0–23)	78 (96.3)	67 (95.7)	215 (92.7)
Mild (24–32)	2 (2.5)	2 (2.9)	10 (4.3)
Moderate (33–36)	1 (1.2)	0 (0.0)	3 (1.3)
Severe psychological impact (> 37)	0 (0.0)	1 (1.4)	4 (1.7)

IES-R, Impact of Events Scale-Revised; STAI, State-Trait Anxiety Inventory

Based on IES-R, few (6%) participants reported experiencing psychological distress related to the landscape fire events (Table 5).

### Concerns about effects of landscape fire smoke exposure on pregnancy

Of the 73 pregnant women who answered the questions related to concerns around their pregnancy, 38% reported having concerns about the effect of landscape fire events on their pregnancy, while 14% were unsure; the main concerns were the effects on their asthma (86%) and unborn baby (75%). Reported actions undertaken in relation to their concerns were predominantly staying indoors/avoiding going outdoors (96%), and keeping windows and doors shut when inside (93%) (Table 6).

**Table 6:** Concerns about the effect of the 2019/20 Australian landscape fire events on pregnancy in women with asthma

Variables	Number (%)
Concerns about the impact of landscape fire events on pregnancy *n* **=** 73	
Yes	28 (38.4)
No	35 (47.9)
Unsure	10 (13.7)
Missing	8
Concerns related to *n* = 28	
Effects on my asthma	24 (85.7)
Effects on my unborn baby	21 (75.0)
Feelings of increased stress	8 (28.6)
Access to care e.g. hospital, midwife, doctor, etc	3 (10.7)
Access to medications	2 (7.1)
Other	1 (3.6)
Actions taken in relation to the concerns *n* = 28	
Stayed indoors/avoided going outdoors	27 (96.4)
Kept windows and doors shut when inside	26 (92.8)
Used an air conditioner in my home	14 (50.0)
Sought medical advice	10 (35.7)
Changed how I used my medications	10 (35.7)
Stockpiled medications	3 (10.7)
Used an indoor air cleaner/purifier	3 (10.7)
Used a face mask	3 (10.7)

## Discussion

This study investigated the impact of the 2019/20 Australian Black summer landscape fires on the physical and mental health of women with asthma, including pregnant and breastfeeding women with asthma. In this sample of women with asthma, a significantly larger proportion (82%) reported general symptoms during the fire period with 41% reporting persistent symptoms approximately 5 months following and higher anxiety scores during the fire period, compared to the period following, indicating that the landscape fire events had a significant physical and mental impact on this group of women. Most participants reported experiencing asthma symptoms and an exacerbation(s) during the fire period, with the majority requiring additional asthma medications. Nearly two in five pregnant women in our sample reported concern about the impact of fire events on their pregnancy, with the majority concerned about their asthma and their unborn baby. Although most participants reported taking specific action(s) to minimise landscape fire smoke exposure, less than half received advice on asthma management or smoke mitigation strategies during the fire period, indicating the need for effective communication of smoke risk mitigation strategies during the fire period.

During the fire period, participants were exposed to a median daily average PM_2.5_ of 16.6 μg/m^3^ and median peak PM_2.5_ of 104.6 μg/m^3^, exceeding the World Health Organization (WHO) 24-hour air quality guideline (15 μg/m^3^) [46]. Although there is mounting evidence supporting an association between landscape fire smoke related PM_2.5_ and respiratory symptoms in the general population, and in adults and children with asthma [10, 12, 13, 47], the impact of prolonged landscape fire smoke exposure on women with asthma has been reported only in one study [11], and none reporting on pregnant or breastfeeding women with asthma. Our findings demonstrate a significant impact of prolonged exposure to landscape fire smoke on the physical and mental health of women with asthma, including pregnant and breastfeeding women, with most participants reporting at least one physical symptom. A study of a high community burden of landscape fire smoke-related symptoms in the Hunter and New England regions in Australia, found that 56% of participants reported symptoms because of smoke exposure [13]. A substantial proportion of participants in our study reported asthma symptoms and exacerbations during the fire period, with a significant number resulting in healthcare utilisation. Notably, most participants attributed their asthma symptoms and exacerbation(s) to smoke exposure. Our study also showed that most pregnant women reported asthma symptoms and exacerbation during the fire period, with 15% of pregnant women reporting they started/increased OCS use for an asthma exacerbation. This could be of particular clinical importance for this group, given the association between asthma exacerbations in pregnancy and poor perinatal outcomes [48]. Similar findings were shown in the 2019/2020 landscape fire survey in Australia, where prolonged landscape fire smoke exposure increased healthcare utilisation by people with asthma [12]. A study of differential respiratory health effects from the 2008 northern California landscape fire has reported that hospitalization and emergency visits for asthma were associated with PM_2.5_ during the fire period [10]. Taken together, these findings highlight the serious effect of exposure to landscape fire smoke on people with asthma and its impact on the healthcare system.

Our study indicated that less than half the cohort, including those who were pregnant or breastfeeding, received information on asthma management or smoke mitigation strategies during the fire period. For those who did receive information, the source varied, and included health professionals, media, social media, and family/friends. Nearly half of pregnant women received information on smoke mitigation strategies from general practitioner while more than half of breastfeeding and neither pregnant nor breastfeeding women received information from News/current affairs stories. However, this study was unable to assess the quality and clarity of the information received. A study of public health messaging during extreme smoke events in Australia has reported that messaging about landscape fire smoke needs to be available from a trusted source with clear links to more detailed information regarding local air quality data along with an explanation of the related health consequences [49]. Therefore, evidence-based, targeted, clear and consistent public health messaging on how women with asthma, including those who are pregnant or breastfeeding, and their health care providers manage and treat health consequences is needed.

Although there has been research investigating the impact of natural disasters on maternal mental health outcomes [21, 50], to our knowledge, this is the first study to assess the impact of prolonged landscape fires on mental health in women with asthma, including women who were pregnant or breastfeeding. Our findings show that participants had higher anxiety during the fire period, compared to approximately 5 months after the fire period. A systematic review and meta-analysis of three studies which investigated the long-term impact of landscape fire on the mental health of Australians demonstrated that 14% of general populations experienced psychological distress after 2–4 years of fire [51]. A study of the health impacts of the prolonged landscape fire smoke exposure in the Australian Capital Territory showed that women, regardless of pregnancy status were more likely to experience negative mental health outcomes such as anxiety and depression during the fire period than men [25]. These findings suggest the need for mental health support and information on effective risk mitigation strategies to vulnerable populations, including women with asthma, during and following landscape fires.

Our study showed that two in five pregnant women with asthma were concerned about the effect of landscape fire events on their pregnancy. Direct comparison between our study and existing literature is difficult given the scarcity of published studies regarding the concern of pregnant women about the effects of fire events on their pregnancy. A study of the experience of pregnant women during Hurricane Maria in the Caribbean reported that pregnant women were worried about losing their pregnancy due to the Hurricane [52]. Our findings highlight the need for emotional support for pregnant women and addressing their concern during landscape fire events, which may have an impact on their pregnancy outcomes.

Our study has limitations. There was a lag of between 3 to 6 months between the end of the landscape fire period and the time of the survey, which may have led to recall bias and misrepresentation in the persistence of symptoms and mental health impacts. Due to a small response rate, there is also the possibility of participation bias where only those women who were more concerned about their health during the fire period might have completed the survey; however, this is a potential limitation common to observational epidemiological studies including cross-sectional studies. Furthermore, availability of exposure data for the study participants was limited by data availability from air quality monitoring stations; as a result, there is a low variation in the exposure data, meaning our study has limited statistical power to determine associations between landscape fire related PM_2.5_ exposure or fire days and health outcomes.

## Conclusion

Despite most women with asthma in this study taking actions to avoid exposure to smoke, the prolonged landscape fire smoke exposure during the 2019/2020 Australian Black Summer fire period had an impact on the physical and mental health of women with asthma, including those who were pregnant or breastfeeding. The findings highlight the need for consistent public health messages, resources and support during the landscape fire events to protect the physical and mental health of women with asthma. Furthermore, longitudinal studies are needed to assess the immediate and long-term impact of landscape fire events on women with asthma, including pregnant and breastfeeding women.

## Supplementary Information

**Additional file 1: Fig. S1.** Landscape fire smoke exposure during the 2019/2020 Australian Black Summer fire period. Exposure was assessed using PM_2.5_ measures from fixed monitoring stations for Sydney Greater Metropolitan Region (New South Wales) (Panel A) and Melbourne (Panel B) regions. Validation of landscape fire activity was obtained from images as seen by the Moderate Resolution Imaging Spectroradiometer (MODIS) Terra Satellite. (A) Population-weighted mean daily PM_2.5_ concentration in the Sydney Greater Metropolitan Region (New South Wales) during the 2019/2020 fire period. (B) Population-weighted mean daily PM_2.5_ concentrations in the Melbourne Region (Victoria) during the 2019/2020 fire period. (C) Landscape fire days identified in the Sydney Greater Metropolitan Region (New South Wales) during the 2019/2020 fire period. (D) Landscape fire days identified in the Melbourne region during the 2019/2020 fire period. (E) Fire hot spots and smoke plumes in the Sydney region as seen by the MODIS Terra satellite on 4 December 2019. (F) Fire hot spots and smoke plumes in the Melbourne region as seen by the MODIS Terra satellite on 14 January 2020. The orange dot indicates fires. **Fig. S2.** Symptoms reported by women with asthma, including pregnant and breastfeeding women, during and following the 2019/20 Australian Black Summer landscape fire period. *indicates statistically significant difference in symptom during versus following the fire period. ** *p* < 0.001, * *p* < 0.05. Table S1. Self-reported asthma symptoms during the fire period experienced by women with asthma, including pregnant and breastfeeding women, during and following the 2019/2020 Australian Black Summer landscape fire period. **Table S2.** Source of information/advice on symptoms, asthma management and minimising exposure to landscape fire smoke reported by women with asthma, including pregnant and breastfeeding women during the 2019/20 Australian landscape fires. **Fig. S3.** Actions taken by women with asthma, including pregnant and breastfeeding women to minimise exposure to landscape fire smoke during the 2019/20 Australian landscape fires. **Fig. S4.** Impact of prolonged smoke exposure from the 2019/20 Australian landscape fires on quality of life in women with asthma, including pregnant and breastfeeding women. **Fig. S5.** Mean anxiety score amongst women with asthma, including pregnant and breastfeeding women, during, and after, the 2019/20 Australian landscape fires. The horizontal dashed line indicates a cut-off point for clinically significant symptoms of anxiety using the STAI-20. Error bars indicate the standard deviation. *indicates statistically significant difference in anxiety during versus following the fire period [*p* < 0.001].

## Data Availability

All data generated during this study are included in the manuscript files.

## Abbreviations

BLT: Breathing for Life Trial
NSW: New South Wales
PM_2.5_: Particulate matter of less than or equal to 2.5μm in diameter
WHO: World Health Organization
