# Supplementary material for: The impact of prolonged landscape fire smoke exposure on women with asthma in Australia
Source: BMC Pregnancy Childbirth. 2022 Dec 8;22:919. doi: 10.1186/s12884-022-05231-8 (PMC9733231; doi:10.1186/s12884-022-05231-8)
Supplement: Supplementary file 1 — Additional file 1: Fig. S1. Landscape fire smoke exposure during the 2019/2020 Australian Black Summer fire period. Exposure was assessed using PM2.5 measures from fixed monitoring stations for Sydney Greater Metropolitan Region (New South Wales) (Panel A) and Melbourne (Panel B) regions. Validation of landscape fire activity was obtained from images as seen by the Moderate Resolution Imaging Spectroradiometer (MODIS) Terra Satellite. (A) Population-weighted mean daily PM2.5 concentration in the Sydney Greater Metropolitan Region (New South Wales) during the 2019/2020 fire period. (B) Population-weighted mean daily PM2.5 concentrations in the Melbourne Region (Victoria) during the 2019/2020 fire period. (C) Landscape fire days identified in the Sydney Greater Metropolitan Region (New South Wales) during the 2019/2020 fire period. (D) Landscape fire days identified in the Melbourne region during the 2019/2020 fire period. (E) Fire hot spots and smoke plumes in the Sydney region as seen by the MODIS Terra satellite on 4 December 2019. (F) Fire hot spots and smoke plumes in the Melbourne region as seen by the MODIS Terra satellite on 14 January 2020. The orange dot indicates fires. Fig. S2. Symptoms reported by women with asthma, including pregnant and breastfeeding women, during and following the 2019/20 Australian Black Summer landscape fire period. *indicates statistically significant difference in symptom during versus following the fire period. ** p < 0.001, * p < 0.05. Table S1. Self-reported asthma symptoms during the fire period experienced by women with asthma, including pregnant and breastfeeding women, during and following the 2019/2020 Australian Black Summer landscape fire period. Table S2. Source of information/advice on symptoms, asthma management and minimising exposure to landscape fire smoke reported by women with asthma, including pregnant and breastfeeding women during the 2019/20 Australian landscape fires. Fig. S3. Actions taken by women with asth [file 12884_2022_5231_MOESM1_ESM.docx]

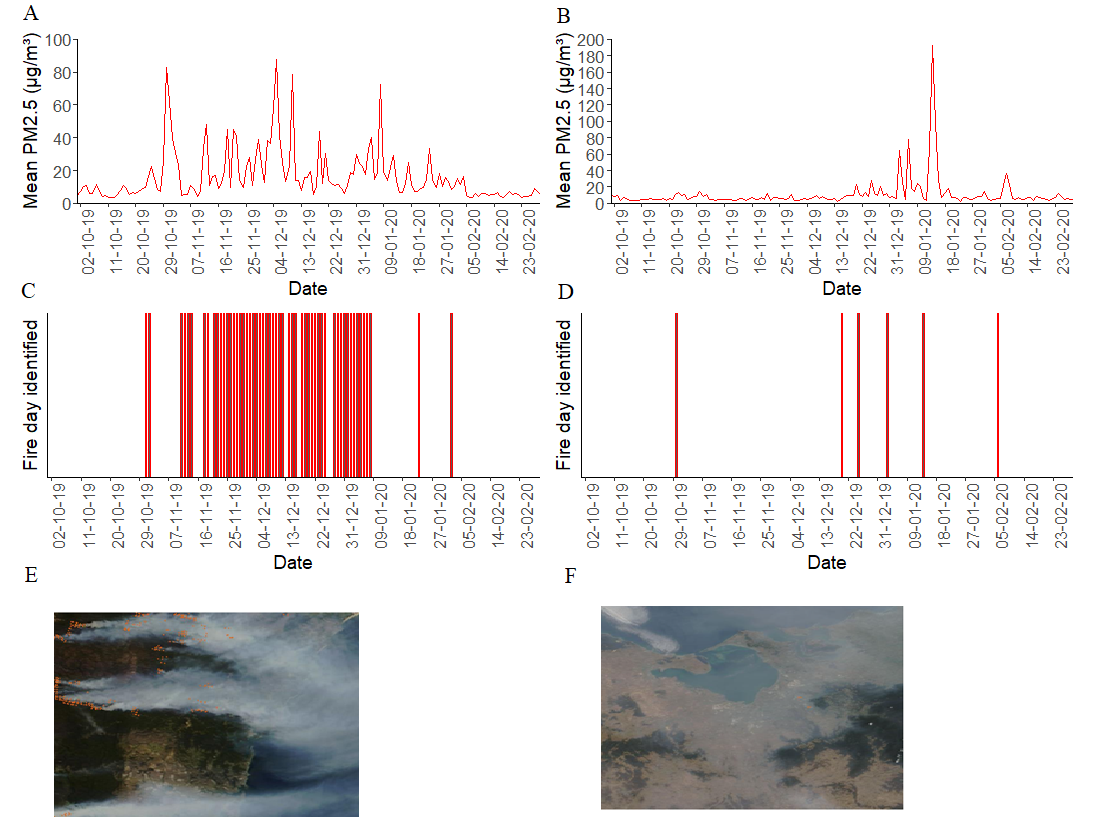


**Fig. S1** Landscape fire smoke exposure during the 2019/2020 Australian Black Summer fire period**.** Exposure was assessed using PM_2.5_ measures from fixed monitoring stations for Sydney Greater Metropolitan Region (New South Wales) (Panel A) and Melbourne (Panel B) regions. Validation of landscape fire activity was obtained from images as seen by the Moderate Resolution Imaging Spectroradiometer (MODIS) Terra Satellite. (A) Population-weighted mean daily PM_2.5_ concentration in the Sydney Greater Metropolitan Region (New South Wales) during the 2019/2020 fire period. (B) Population-weighted mean daily PM_2.5_ concentrations in the Melbourne Region (Victoria) during the 2019/2020 fire period. (C) Landscape fire days identified in the Sydney Greater Metropolitan Region (New South Wales) during the 2019/2020 fire period. (D) Landscape fire days identified in the Melbourne region during the 2019/2020 fire period. **(E)** Fire hot spots and smoke plumes in the Sydney region as seen by the MODIS Terra satellite on 4 December 2019. **(F)** Fire hot spots and smoke plumes in the Melbourne region as seen by the MODIS Terra satellite on 14 January 2020. The orange dot indicates fires.


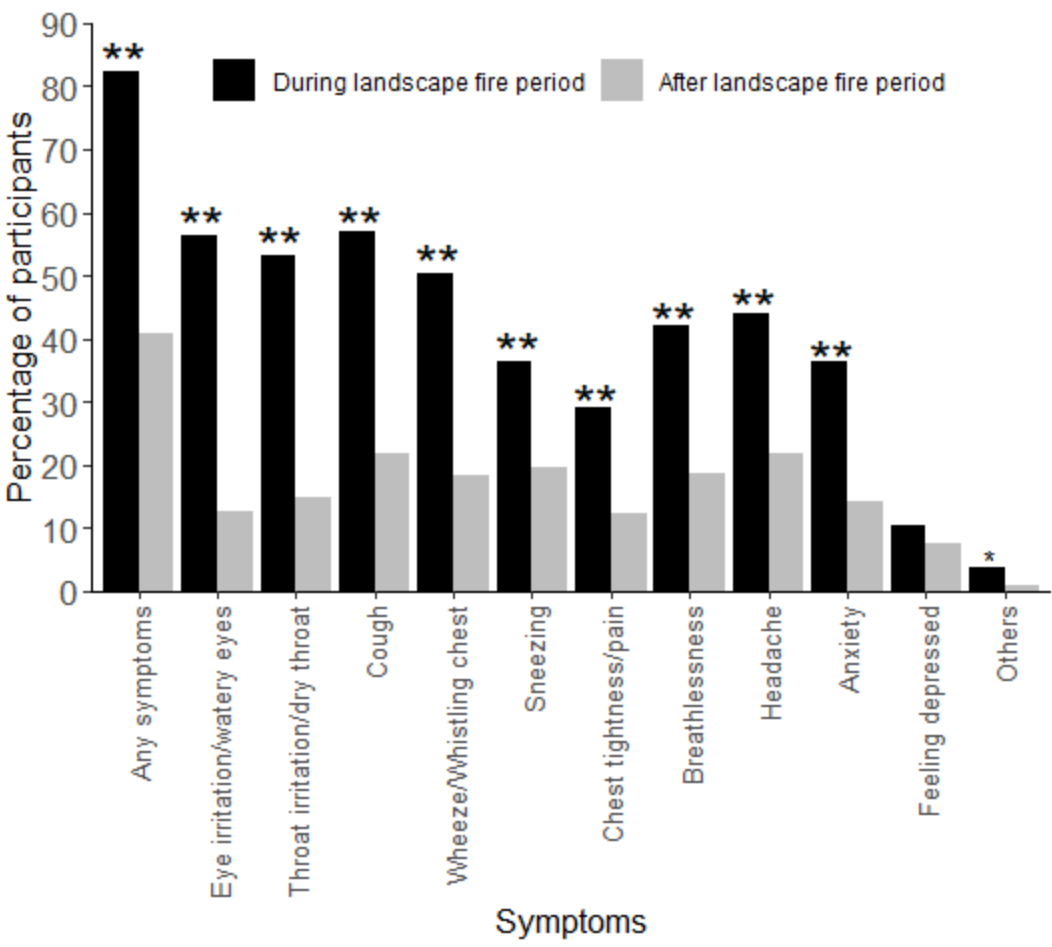


**Fig. S2** Symptoms reported by women with asthma, including pregnant and breastfeeding women, during and following the 2019/20 Australian Black Summer landscape fire period. *indicates statistically significant difference in symptom during versus following the fire period. ** p <0.001, * p < 0.05.

**Table S1** Self-reported asthma symptoms during the fire period experienced by women with asthma, including pregnant and breastfeeding women, during and following the 2019/2020 Australian Black Summer landscape fire period.

| **Variable** | **value** |
| --- | --- |
| Experienced asthma symptoms during the landscape fire period, n (%) | 326 (85.1) |
| Exposure to smoke was the main reason for asthma symptoms, n (%) |  |
| Yes | 277 (85.0) |
| No | 7 (2.1) |
| Don’t know/Unsure | 39 (12.0) |
| Not exposed to landscape fire | 3 (0.9) |
| Asthma exacerbation | 328 (85.6) |
| Types of asthma exacerbation, n (%) |  |
| Emergency department | 15 (3.9) |
| Unscheduled doctor visit | 105 (27.4) |
| Start/increase of OCS at least 3 days | 77 (20.1) |
| IV corticosteroids | 5 (1.3) |
| Increased reliever use | 313 (81.7) |
| Increased preventer dose/frequency | 209 (54.6) |
| Number of times, median (Q1, Q3) |  |
| Emergency department | 1 (1,1) |
| Unscheduled doctor visit | 2 (1,3) |
| Start/increase of OCS ≥3 days | 2 (1,3) |
| IV corticosteroids | 1 (0,1) |
| Thinks smoke from the landscape fires was the main reason for the exacerbation, n (%) | 285 (86.9) |
| Not exposed | 8 (2.4) |

**Table S2** Source of information/advice on symptoms, asthma management and minimising exposure to landscape fire smoke reported by women with asthma, including pregnant and breastfeeding women during the 2019/20 Australian landscape fires.

| **Action taken** | **n (%)** |
| --- | --- |
|  |  |
| Sought health advice from a health professional for symptoms | 154/315 (48.9) |
| General practitioner | 118 (37.5) |
| Pharmacist | 56 (17.8) |
| Other medical professional | 19 (6.0) |
| Emergency department | 9 (2.8) |
| 24-hour health advice hotline | 8 (2.5) |
| Hospital inpatient | 2 (0.6) |
| Took time off work because of the symptoms | 85 (27.0) |
| Landscape fire was the main reason for any symptoms |  |
| Yes | 261 (82.9) |
| No | 15 (4.8) |
| Do not know/unsure | 35 (11.1) |
| Not exposed to wildfire | 4 (1.3) |
| Advice on asthma management | 148 (38.6) |
| General practitioner | 107 (72.3) |
| Pharmacist | 34 (23.0) |
| News/current affairs stories | 30 (20.3) |
| Respiratory/asthma specialist | 19 (12.8) |
| Social media | 17 (11.5) |
| Nurse | 12 (8.1) |
| Family/friends | 9 (6.1) |
| Health department | 8 (5.4) |
| Asthma Australia | 7 (4.7) |
| Midwife/Obstetrician | 6 (4.1) |
| Others* | 12 (8.1) |
| Received advice about how to avoid or minimise exposure to landscape fire smoke | 174 (45.4) |
| News/current affairs stories | 88 (50.6) |
| General practitioner | 69 (39.7) |
| Social media | 69 (39.7) |
| Family/friends | 30 (17.2) |
| Health department | 29 (16.7) |
| Pharmacist | 20 (11.5) |
| Respiratory/asthma specialist | 12 (6.9) |
| Asthma Australia | 11 (6.3) |
| Nurse | 7 (4.0) |
| Midwife/Obstetrician | 5 (2.8) |
| Others* | 21 (12.1) |

* clinical research assistance (nurse), support group, 24-hour health advice hotline, other medical professionals; NA, not applicable


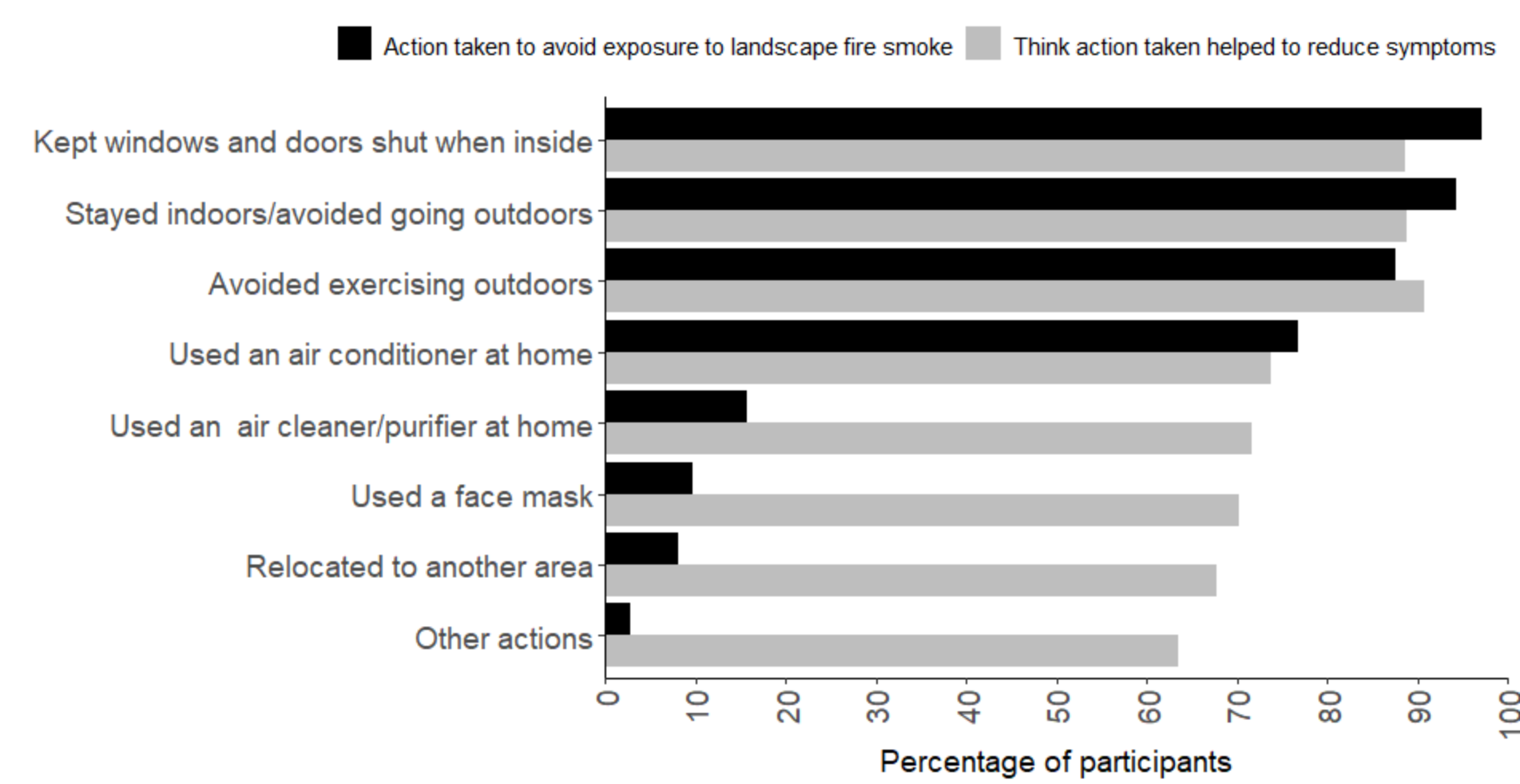
 **Fig. S3** Actions taken by women with asthma, including pregnant and breastfeeding women to minimise exposure to landscape fire smoke during the 2019/20 Australian landscape fires.


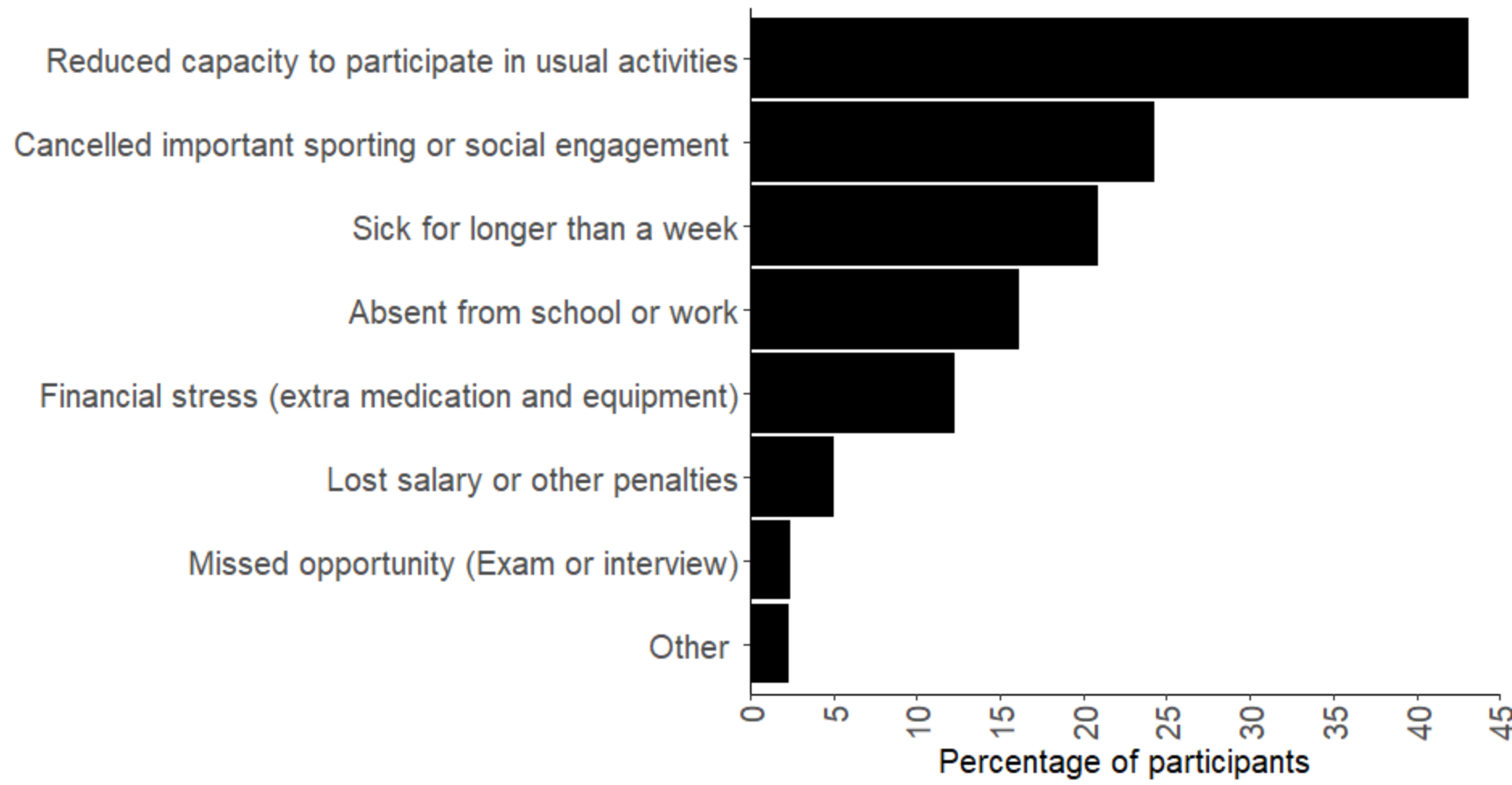


**Fig. S4** Impact of prolonged smoke exposure from the 2019/20 Australian landscape fires on quality of life in women with asthma, including pregnant and breastfeeding women.


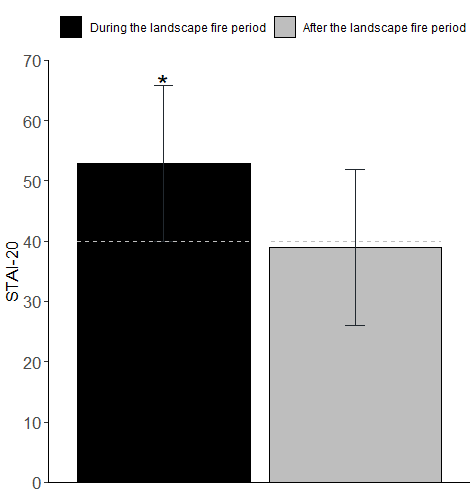


**Fig. S5** Mean anxiety score amongst women with asthma, including pregnant and breastfeeding women, during, and after, the 2019/20 Australian landscape fires. The horizontal dashed line indicates a cut-off point for clinically significant symptoms of anxiety using the STAI-20. Error bars indicate the standard deviation. *indicates statistically significant difference in anxiety during versus following the fire period [p<0.001].
